# Supplementary material for: Efficacy of Deep TMS with the H1 Coil for Anxious Depression
Source: J Clin Med. 2022 Feb 15;11(4):1015. doi: 10.3390/jcm11041015 (PMC8879826; doi:10.3390/jcm11041015)
Supplement: Supplementary file 1 [file jcm-11-01015-s001.zip › jcm-1573286-supplementary.pdf]

Journal of
Clinical Medicine

Supplementary Material

Meta-analyses of pharmacotherapies: Anxious Depression

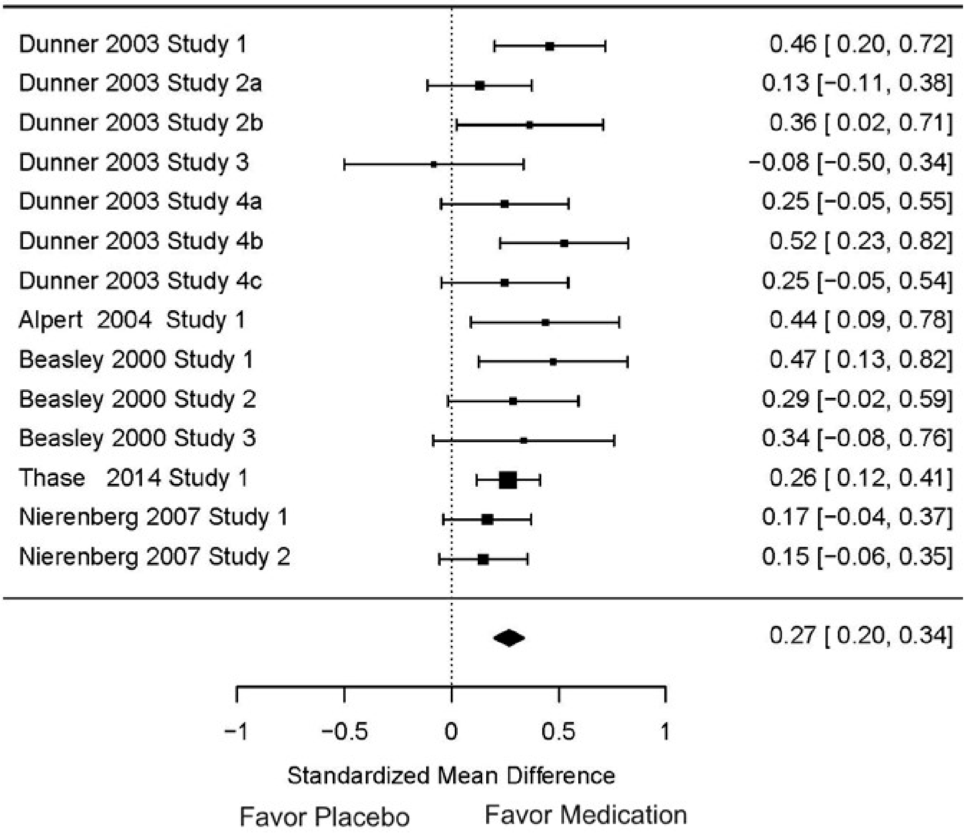

Figure S1. Forest plot of anxiolytic medication trials for anxious depression that reported the HDRS-A/S factor scale.

Additional figure notes:
A literature search was used to locate trials of anxious depression where the HDRS A/S scale was used to track anxiety changes.

Details and references of each trial are as follows:
Dunner 2003: Study 1-Duloxetine 60mg vs. placebo [1]
Dunner 2003: Study 2-Duloxetine 60mg vs. placebo [1]
Dunner 2003: Study 2-Duloxetine 120mg vs. placebo [1]
Dunner 2003: Study 3-Fluoxetine 20mg vs. placebo [1]
Dunner 2003: Study 4-Duloxetine 40mg vs. placebo [1]
Dunner 2003: Study 4-Duloxetine 80mg vs. placebo [1]
Dunner 2003: Study 4-Paroxetine 20mg vs. placebo [1]
Alpert 2004: Study 1-Gepirone ER 20-80mg vs. placebo [2]
Beasley 2000: Study 1-Fluoxetine 20mg vs. placebo [3]
Beasley 2000: Study 2-Fluoxetine 20mg vs. placebo [3]
Beasley 2000: Study 3-Fluoxetine 20mg vs. placebo [3]
Thase 2014: Study 1-Vilazodone 40mg vs. placebo [4]
Nierenberg 2007: Study 1-Duloxetine 60mg vs. placebo [5]
Nierenberg 2007: Study 2-Escilatopram 10mg vs. placebo [5]

Meta-analyses of pharmacotherapies: Generalized anxiety disorder (GAD)

	Statistics for each study						
	Std diff in means	Standard error	Variance	Lower limit	Upper limit	Z-Value	p-Value
Ven XR 75 mg-1	-0.171	0.146	0.021	-0.457	0.114	-1.175	0.240
Ven XR 150 mg-1	-0.264	0.147	0.021	-0.551	0.023	-1.801	0.072
Ven XR 225 mg-1	-0.324	0.147	0.022	-0.613	-0.035	-2.201	0.028
Ven XR 75 mg-2	-0.332	0.141	0.020	-0.608	-0.055	-2.353	0.019
Ven XR 150 mg-2	-0.212	0.141	0.020	-0.488	0.064	-1.503	0.133
Dul 60 mg	-0.498	0.110	0.012	-0.713	-0.283	-4.541	0.000
Dul 120 mg	-0.466	0.109	0.012	-0.680	-0.252	-4.269	0.000
Dul 60-120 mg-1	-0.246	0.111	0.012	-0.464	-0.028	-2.214	0.027
Dul 60-120 mg-2	-0.301	0.112	0.013	-0.520	-0.082	-2.689	0.007
Par 20 mg	-0.328	0.105	0.011	-0.534	-0.122	-3.124	0.002
Par 40 mg	-0.291	0.104	0.011	-0.494	-0.088	-2.806	0.005
Par 20-50 mg-1	-0.258	0.112	0.012	-0.477	-0.039	-2.314	0.021
Par 20-50 mg-2	-0.102	0.105	0.011	-0.308	0.104	-0.972	0.331
Lex 10-20 mg-1	-0.282	0.127	0.016	-0.530	-0.034	-2.228	0.026
Lex 10-20 mg-2	-0.270	0.120	0.014	-0.505	-0.035	-2.253	0.024
Lex 10-20 mg-3	-0.525	0.116	0.013	-0.752	-0.297	-4.518	0.000
Overall	-0.309	0.030	0.001	-0.368	-0.251	-10.392	0.000

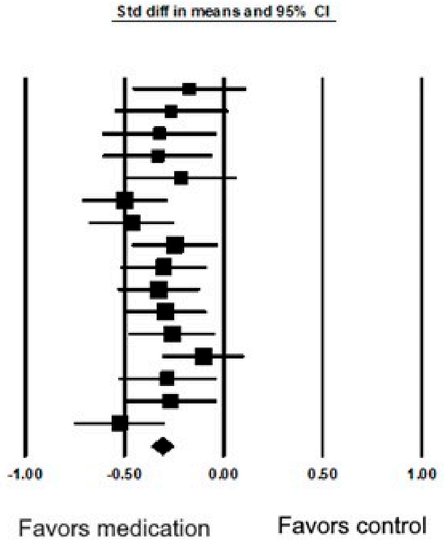

Figure S2. Forest plot of anxiolytic medication trials of generalized anxiety disorder (GAD). Data was gathered from the FDA Drug database of NDAs for Venlafaxine (Effexor), Duloxetine (Cymbalta), Paroxetine (Paxil) and Escitalopram (Lexapro/Cipralex).

Additional figure notes

For all of these studies, the anxiety rating scale was the HAM-A scale.
Details of the ordered studies are as follows:
Venlafaxine 75mg, Study 1, n=189; NDA # 020699
Venlafaxine 150mg, Study 1, n=188; NDA # 020699
Venlafaxine 225mg, Study 1, n=187; NDA # 020699
Venlafaxine 75mg, Study 2, n=204; NDA # 020699
Venlafaxine 150mg, Study 2, n=203; NDA # 020699
Duloxetine 60mg, Study 1, n=343; NDA # 021427
Duloxetine 120mg, Study 1, n=345; NDA # 021427
Duloxetine 60-120mg, Study 2, n=327; NDA # 021427
Duloxetine 60-120mg, Study 3, n=323; NDA # 021427
Paroxetine 20mg, Study 641, n=368; NDA # 020031
Paroxetine 40mg, Study 641, n=377; NDA # 020031
Paroxetine 20-50mg, Study 642, n=324; NDA # 020031
Paroxetine 20-50mg, Study 637, n=364; NDA # 020031
Lexapro (Escitalopram) 10-20mg, Study 1, n=252; NDA # 021323 & 021365
Lexapro (Escitalopram) 10-20mg, Study 2, n=281; NDA # 021323 & 021365
Lexapro (Escitalopram) 10-20mg, Study 1, n=307; NDA # 021323 & 021365

References

37. Dunner DL, Goldstein DJ, Mallinckrodt C, Lu Y, Detke MJ. Duloxetine in treatment of anxiety symptoms associated with depression. Depress Anxiety. 2003;18(2):53-61. doi: 10.1002/da.10122. PMID: 12964171.
38. Alpert JE, Franznick DA, Hollander SB, Fava M. Gepirone extended-release treatment of anxious depression: evidence from a retrospective subgroup analysis in patients with major depressive disorder. J Clin Psychiatry. 2004 Aug;65(8):1069-75. PMID: 15323591.
39. Bandelow B, Andersen HF, Dolberg OT. Escitalopram in the treatment of anxiety symptoms associated with depression. Depress Anxiety. 2007;24(1):53-61. doi: 10.1002/da.20141. PMID: 16937393.
40. Thase ME, Chen D, Edwards J, Ruth A. Efficacy of vilazodone on anxiety symptoms in patients with major depressive disorder. Int Clin Psychopharmacol. 2014 Nov;29(6):351-6.

41. Nierenberg AA, Greist JH, Mallinckrodt CH, Prakash A, Sambunaris A, Tollefson GD, Wohlreich MM. Duloxetine versus escitalopram and placebo in the treatment of patients with major depressive disorder: onset of antidepressant action, a non-inferiority study. Curr Med Res Opin. 2007 Feb;23(2):401-16.
